# Molecular chaperones involved in mitochondrial iron–sulfur protein biogenesis

**DOI:** 10.1007/s00775-017-1504-x

**Published:** 2017-11-09

**Authors:** Rafal Dutkiewicz, Malgorzata Nowak

**Affiliations:** Intercollegiate Faculty of Biotechnology, University of Gdansk and Medical University of Gdansk, Abrahama 58, 80-307 Gdańsk, Poland

**Keywords:** J-domain proteins, Hsp70, Iron–sulfur clusters, Mitochondria, Yeast

## Abstract

Iron–sulfur (FeS) clusters are prosthetic groups critical for the function of many proteins in all domains of life. FeS proteins function in processes ranging from oxidative phosphorylation and cofactor biosyntheses to DNA/RNA metabolism and regulation of gene expression. In eukaryotic cells, mitochondria play a central role in the process of FeS biogenesis and support maturation of FeS proteins localized within mitochondria and in other cellular compartments. In humans, defects in mitochondrial FeS cluster biogenesis lead to numerous pathologies, which are often fatal. The generation of FeS clusters in mitochondria is a complex process. The [2Fe–2S] cluster is first assembled on a dedicated scaffold protein (Isu1) by the action of protein factors that interact with Isu1 to form the “assembly complex”. Next, the FeS cluster is transferred onto a recipient apo-protein. Genetic and biochemical evidence implicates participation of a specialized J-protein co-chaperone Jac1 and its mitochondrial (mt)Hsp70 chaperone partner, and the glutaredoxin Grx5 in the FeS cluster transfer process. Finally, various specialized ISC components assist in the generation of [4Fe–4S] clusters and cluster insertion into specific target apoproteins. Although a framework of protein components that are involved in the mitochondrial FeS cluster biogenesis has been established based on genetic and biochemical studies, detailed molecular mechanisms involved in this important and medically relevant process are not well understood. This review summarizes our molecular knowledge on chaperone proteins’ functions during the FeS protein biogenesis.

## Introduction

**Fig. 1:**
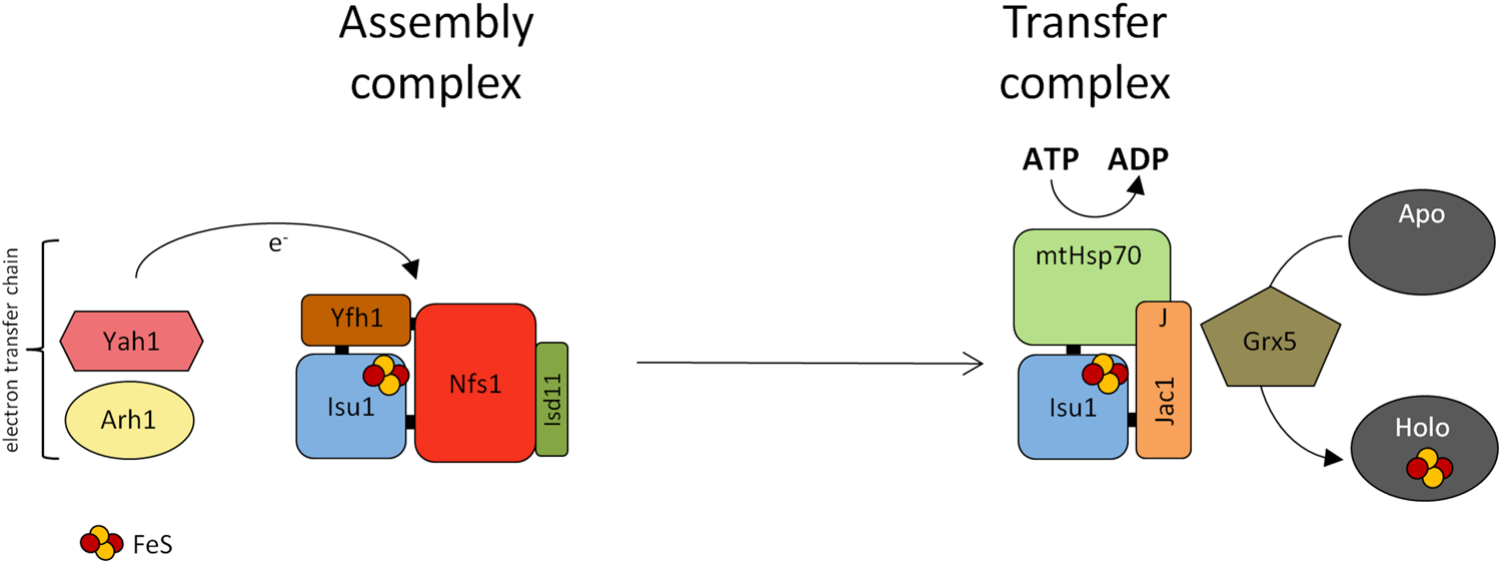
Ordered maturation of FeS clusters within mitochondria. Yfh1 binds the pre-formed Isu1–Nfs1(Isd11) complex facilitating FeS cluster synthesis. Other factors needed for cluster assembly include: yeast ferredoxin Yah1, which is functionally coupled with ferredoxin reductase Arh1, to provide electrons required for sulfur reduction. Jac1 displaces holo-Isu1 from the assembly complex to form the holo-Isu1–Jac1 complex. Ssq1 binds the PVK motif of holo-Isu1 in complex with Jac1. J-domain (*J*) of Jac1 stimulates the ATPase activity of Ssq1 facilitating FeS cluster transfer to the recipient apoprotein

Biogenesis of mitochondrial iron–sulfur (FeS) proteins requires the interaction of multiple proteins with the highly conserved 14-kDa scaffold protein Isu1, on which clusters are built prior to their transfer to recipient proteins. The assembly process of FeS cluster on Isu1 involves the interaction of molecular scaffold with both Nfs1, the cysteine desulfurase serving as a sulfur donor, and the yeast frataxin homolog (Yfh1) serving as a regulator of desulfurase activity and/or iron donor. The transfer process requires Hsp70 chaperone system [1–3] (Fig. 1).

Biochemical data indicate that the transfer of a FeS cluster requires a typical Hsp70 reaction cycle. The specialized J-protein called HscB in *Escherichia coli* and Jac1 in *Saccharomyces cerevisiae* binds the FeS scaffold using its C-terminal domain. Next, HscB/Jac1 interacts with Hsp70 via the N-terminal J-domain, which is highly similar to the J-domains of other J-proteins. This results in stimulation of Hsp70’s ATPase activity that promotes its interaction with the FeS scaffold and transfer of the cluster to next targets [4, 5].

The mitochondrial pathway of FeS cluster biogenesis was inherited from bacteria, including the involvement of Hsp70 chaperone machinery in the process. However, the evolutionary analysis revealed that while mitochondria inherited the J-protein co-chaperone from bacterial ancestors, it did not inherit the specialized Hsp70 HscA. As a consequence, most eukaryotes, including fungi distantly related to *S. cerevisiae*, plants, animals and humans, have a single multifunctional Hsp70 in mitochondria. This multifunctional Hsp70 (called Ssc1 in fungi) is orthologous to DnaK, a general chaperone of *E. coli*. Ssc1 not only functions in general protein folding and import of proteins into the mitochondrial matrix but also, unlike DnaK, functions in FeS biogenesis [6, 7].

The system described above functions in most eukaryotes. However, *S. cerevisiae* and closely related fungal species express an additional mtHsp70, called Ssq1, which is specialized in FeS cluster biogenesis. Similarly to multifunctional mtHsp70, it functions with Jac1, but in contrast to mtHsp70, its client-binding specificity is restricted to the FeS cluster scaffold. Ssq1 evolved via an ancestral gene duplication of mtHsp70. In post-duplication species Jac1 coevolved with Ssq1, acquiring structural changes within its J-domain in the process. The altered J-domain became highly specific for Ssq1. Thus, Ssq1 and Jac1 form a highly specialized Hsp70 machine dedicated solely to FeS protein biogenesis. However, all evidence to date indicates that the mode of action of this newly evolved machine is the same as the one utilizing the multifunctional mtHsp70 [6, 8, 9].

In this review, we summarize the current knowledge on molecular chaperones which function in FeS protein biogenesis in mitochondria with an emphasis on the description of the molecular role and functional interactions with components of mitochondrial iron–sulfur cluster (ISC) assembly machinery.

## FeS protein biogenesis requires the Hsp70 system

FeS clusters are attached to the polypeptide primarily via cysteinate iron ligation and constitute one of the most ubiquitous and structurally and functionally diverse classes of biological prosthetic groups [10]. The most prevalent clusters are the rhomboid [2Fe–2S] and the cubane [4Fe–4S], yet more complex forms have been characterized [11, 12]. Biochemical “utility” of FeS clusters as a redox partner is based on their ability to bind and release electrons [10]. This unique chemical property allows the proteins containing FeS clusters to perform a variety of metabolic functions ranging from the obvious participation in electron transfer reactions, reduction of sulfur and nitrate, nitrogen assimilation and cofactor biosyntheses, to less obvious roles in biogenesis of ribosomes, and DNA repair [10, 13, 14]. It is believed that proteins containing FeS clusters have evolved when the oxygen concentration in the Earth’s atmosphere was low. Later, when the oxygen appeared via photosynthesis, organisms adapted to protect FeS clusters against oxidative stress. Sensitivity of FeS clusters to the presence of oxygen is a factor that makes their studies difficult. For example in vitro reconstitution of FeS clusters on proteins requires strictly anaerobic conditions [15].

FeS clusters biogenesis is a complex and coordinated process that involves a large number of dedicated proteins [16, 17]. The maturation of bacterial FeS proteins has been intensely studied in *Escherichia coli* and the azototrophic (nitrogen-fixing) *Azotobacter vinelandii*. Studies have identified three different systems for the biogenesis of bacterial FeS proteins: the NIF system, for specific maturation of nitrogenase in azototrophic bacteria; and the ISC assembly and SUF systems, for the generation of housekeeping FeS proteins under normal and oxidative-stress conditions, respectively [10, 18–20].

In eukaryotic cells, mitochondria play an essential role in the maturation of FeS cluster containing proteins functioning in all subcellular compartments. Although mitochondria inherited many proteins involved in FeS cluster biogenesis from their bacterial ancestors during evolution, some key players were replaced by eukaryote-specific proteins, one of the examples can be mtHsp70 which needs the cooperation with nucleotide exchange factor (NEF)—Mge1. In eukaryotic cells, the majority of proteins involved in FeS cluster biogenesis are essential for cell viability underscoring the biological importance of FeS cluster containing proteins [21–26].

In both mitochondrial and bacterial FeS cluster biogenesis systems, a highly conserved 14-kDa protein (termed Isu1 in *S. cerevisiae*) plays a central role serving as a scaffold for de novo cluster assembly and as a platform for cluster transfer onto recipient apoproteins (Fig. 1). Both the assembly and transfer steps of FeS cluster biogenesis involve the interaction of Isu1 with dedicated protein factors. In *S. cerevisiae*, Isu is encoded by the two closely related and functionally exchangeable paralogous genes, *ISU1* and *ISU2*. Because *ISU1* plays the major role due to its higher expression level [27, 28], in this review we will focus exclusively on this paralogue.

De novo assembly of FeS clusters requires that Isu1 interact with the cysteine desulfurase Nfs1, which delivers sulfur, and the yeast frataxin ortholog, Yfh1, which is a putative iron donor and/or positive regulator of Nfs1 enzymatic activity. Yfh1 and Nfs1 interact with each other and with Isu1, thereby forming the core FeS cluster assembly complex, which constitutes the structural and functional unit responsible for cluster synthesis within the scaffold protein. In eukaryotes, but not in bacteria, Nfs1 functions as a stable heterodimer in complex with a small accessory protein Isd11 [referred to as Nfs1(Isd11) in this review]. Isd11 is proposed to both stabilize Nfs1 and regulate its catalytic activity [29–32]. Nfs1(Isd11) releases sulfur from cysteine by generating a Nfs1-bound persulfide that is then transferred to one of the three conserved cysteine residues of Isu1. While the function of Nfs1(Isd11) is well established, the role of Yfh1 in FeS cluster biosynthesis is being debated. Recent data support the view that the function of Yfh1 is directly related to its role as a component of the FeS cluster assembly complex, but whether it serves as an iron donor, activator of cysteine desulfurase, or both is unresolved. Besides proteins forming the core assembly complex (Isu1–Nfs1(Isd11)–Yfh1), additional factors are required for de novo FeS cluster synthesis. Namely, mitochondrial ferredoxin reductase Arh1 and ferredoxin Yah1 were reported to form an electron transfer chain that supplies electrons for the reduction of the persulfide sulfur (S^0^) to the sulfide (S^2−^) present in the FeS cluster [27, 33–36]. Currently, the main question is which component of ISC machinery is the interaction partner for ferredoxin, because conflicting models exist in the literature concerning this interaction. (1) First, it was shown using a combination of biophysical tools, mutagenesis and computer modeling, that bacterial ferredoxin (Fdx) replaces CyaY, the bacterial ortholog of frataxin, interacting with IscS and IscU [37]. (2) Second, in vitro analysis of FeS cluster synthesis on eukaryotic scaffold Isu1 suggests that Yah1 interacts with Isu1 in the context of the intact assembly complex, forming a ternary Yah1–Isu1–Nfs1(Isd11)–Yfh1 complex [38]. (3) Finally, NMR analysis and cross-linking experiments suggest that *E. coli* ferredoxin Fdx binds directly to IscS, competing with IscU [39].

**Fig. 2:**
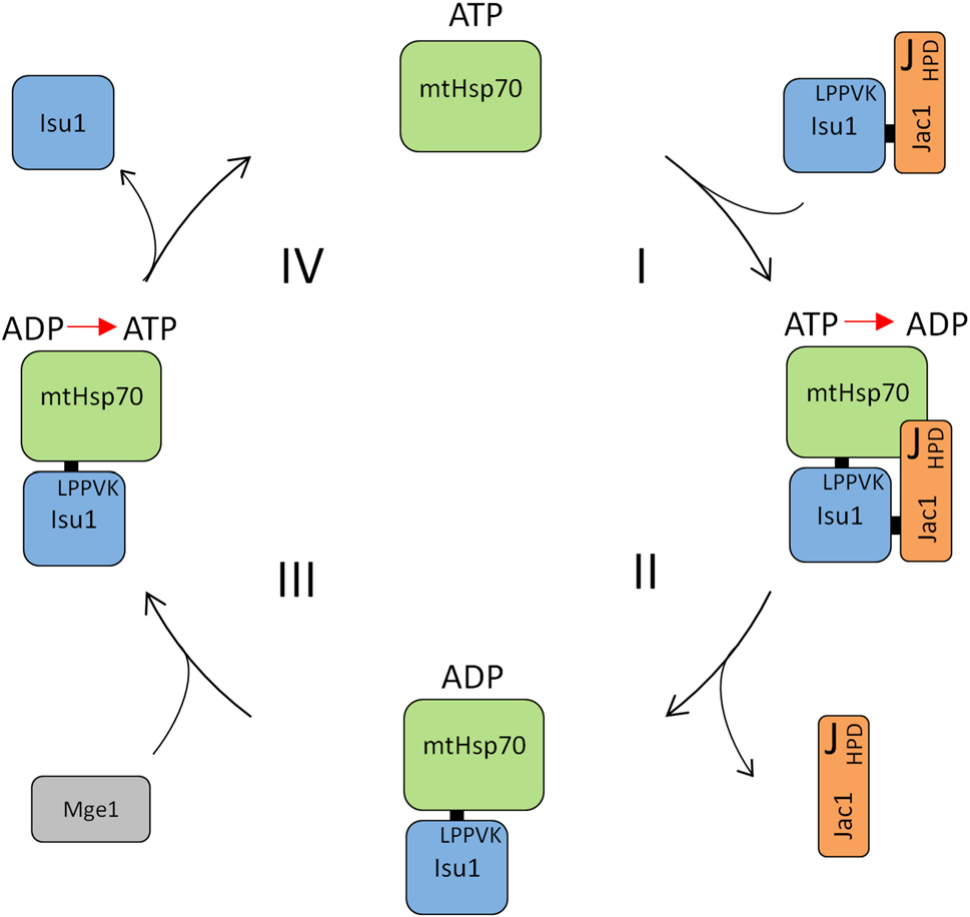
ATPase cycle of chaperone system involved in FeS biogenesis. J-type co-chaperone, Jac1, forms a complex with substrate, Isu1 and facilitate its delivery to mtHsp70. (I) The stability of the mtHsp70-protein substrate interaction depends on the conformation of the chaperone, which is regulated by the bound nucleotide. When ATP is bound, binding of substrate is relatively unstable. Therefore, ATP hydrolysis converts mtHsp70 to the form which has a relatively stable interaction with Isu1. Jac1 interacts directly with mtHsp70 and increases the stability of the mtHsp70-Isu1 interaction by stimulating the ATPase activity of mtHsp70 (II). Exchange of ADP for ATP results in dissociation of the bound Isu1. Exchange of ADP for ATP within mtHsp70 is supported through the cooperation with a nucleotide exchange factor (NEF). In yeast mitochondria, the only known NEF is Mge1 (III). Binding of ATP results in dissociation of the bound Isu1 and makes mtHsp70 ready for the next cycle (IV) [56]

After the initial phase of FeS cluster synthesis on Isu1, the cluster is released from the scaffold, transferred to target apoproteins and inserted into the polypeptide chain. The release and transfer of the Isu1-bound FeS cluster is executed by the Hsp70 chaperone system. A mitochondrial Hsp70 molecular chaperone system is central to the transfer process of FeS cluster from Isu1 to the recipient proteins. In *Saccharomyces cerevisiae,* it is composed of the Hsp70 Ssq1 and its J protein co-chaperone Jac1, as well as the nucleotide release factor Mge1. Isu1 is well-defined client protein for the Jac1/Ssq1 pair [5]. Similarly, in *Escherichia coli*, IscU is the client for the Hsp70 HscA and J-protein HscB pair, but HscA does not appear to require a NEF (nucleotide exchange factor) for exchange of ADP for ATP during the reaction cycle, as its interaction with nucleotide is intrinsically transient [4]. Moreover, dislocation of FeS from the Isu1 scaffold protein to target apoprotein requires the monothiol glutaredoxin Grx5 which participates in the FeS cluster transfer through the interaction with mtHsp70. It seems likely that after FeS cluster is assembled on Isu1, it is then transferred to Grx5 by the assistance of the chaperones. The transient character of FeS cluster binding and the ability to transfer it to target apoproteins have been taken to suggest that Grx5 assists FeS protein maturation by serving as a transient FeS cluster binding site before the cluster is inserted into apoproteins [40, 41] (Fig. 2).

## Interactions of chaperones that make the Hsp70 system work during FeS protein biogenesis

The overall structure of Hsp70s is highly conserved [5]. They have two domains: an N-terminal nucleotide binding domain (NBD) of ~ 44 kDa, with two lobes forming a deep cleft in which adenine nucleotide binds and a C-terminal substrate binding domain (SBD) of ~ 26 kDa (PBD ID: 2KHO). The peptide-binding site, which contacts five contiguous residues of a client polypeptide, is within a β-sandwich structure; an α helix folds back upon the sandwich forming a “lid” over the binding pocket [42]. The two domains are joined by a flexible linker, which plays an important role in modulating the interaction between them [43–45]. Interdomain communication [46] is critical as the adenosine diphosphate (ADP)- and ATP-bound states have profoundly different effects on client protein binding. In the ADP-state, Hsp70s exhibit relatively stable binding; in the ATP-state, binding of peptide is unstable. However, in vivo, the ATP-bound Hsp70 initiates productive interactions with a client polypeptide, because the on-rate (but also the off-rate) is very rapid, in the order of milliseconds. ATP hydrolysis converts Hsp70 to the ADP-state, with a client protein off-rate in the order of minutes leading to rather stable binding. Exchange of ADP for ATP results in dissociation of the bound peptide/polypeptide [42]. Thus, although the molecular basis of interdomain communication remains elusive, it is clear that this interaction is fundamental to the chaperone activity of Hsp70, as the nucleotide bound to the ATPase domain profoundly affects the character of the SBD’s interaction with client proteins [47, 48] (Fig. 2).

Under physiological conditions, when ATP concentrations are typically high, substrate protein interacts with Hsp70 in the ATP conformation. This interaction is stabilized upon hydrolysis of ATP. The cycle of interaction is completed when ADP is replaced by ATP and the substrate is released. Thus, stimulation of the ATPase activity of Hsp70 is essential for stabilization of the Hsp70–substrate interaction. However, the rate of the intrinsic ATPase activity of Hsp70 is low. ATPase activity is stimulated both by interaction of the substrate in the substrate binding cleft and J-protein co-chaperone interaction with the ATPase domain.

Various J-protein/Hsp70 systems present in the eukaryotic cell are involved in many critical processes including protein folding, refolding of protein aggregates, protein trafficking across biological membranes and rearrangement of protein complexes. Yet, according to our current knowledge, the fundamental biochemical mechanism used by all these systems is the same: (1) binding of a short, usually hydrophobic, polypeptide segment on a surface of client protein by Hsp70 and stimulation of Hsp70’s ATPase activity by its partner J-protein, which stabilizes the Hsp70-client interaction. All J-proteins contains a J-domain, responsible for Hsp70’s ATPase stimulation. Some J-proteins also bind client protein, “delivering” it to Hsp70.

**Fig. 3:**
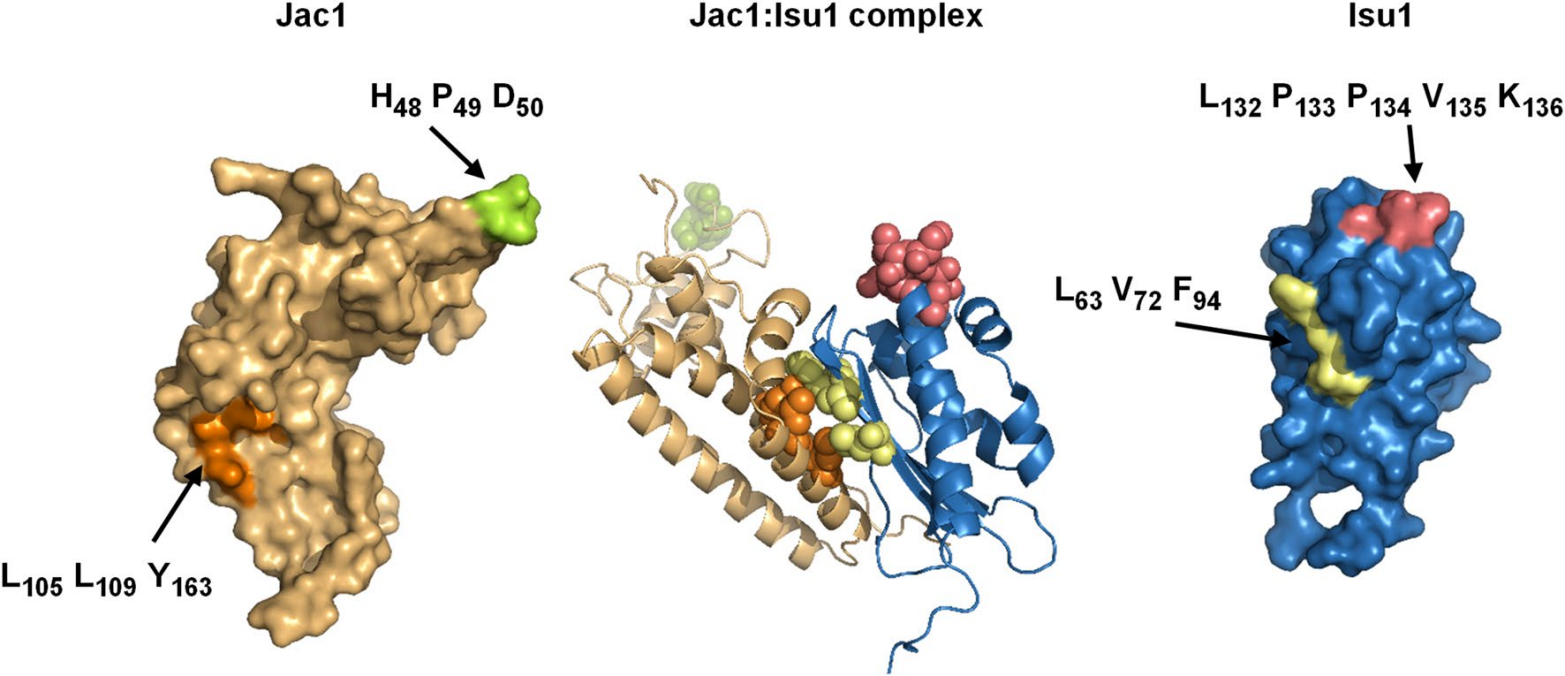
Model of the Jac1–Isu1 complex (center panel) on the basis of in silico docking of the Jac1 protein crystal structure (PDB code 3UO3, left panel) and homology model of the Isu1 structure (right panel). Residues of Jac1 and Isu1 implicated in their interaction are highlighted [51]

The overall structure, obtained for yeast Jac1 resembles structures of its orthologs: HscB from *E. coli* [49] (PDB ID: 1FPO), HscB from *Vibrio cholerae* (PDB ID: 3HHO), and hHSC20 from *Homo sapiens* [50] (PDB ID: 3BVO) with which it shares 29, 31, and 28% sequence identity, respectively (Fig. 3). The structure of Jac1 is L-shaped and consists of two distinct α-helical domains: the N-terminal J-domain (residues 11–84) and the C-terminal Isu binding C-domain (residues 101–184). These two domains are connected by a linker (residues 85–100). The core of the J-domain contains three α-helices, with helices H2 and H3 comprising an antiparallel coiled coil connected by a loop with the conserved J domain histidine:proline:aspartic acid (HPD) signature motif [51] (Fig. 3). The universal function of J-domains of J proteins is stimulation of the ATPase activity of Hsp70s, an activity that requires a conserved HPD tripeptide and results in stabilization of an interaction between an Hsp70 and its client protein. Such activity is critical for Jac1 function, as alteration of HPD to three alanines (AAA) profoundly decreases the ability of Jac1 to stimulate Ssq1 ATPase activity. The AAA mutant protein is unable to rescue a *JAC1* deletion cell which is inviable [52, 53].

All Jac1 orthologues present a remarkable structural conservation of the J- and the C-terminal domains. These regions in specialized co-chaperones from bacteria and yeast were found to be crucial for the interaction with the Hsp70 chaperone partner and the respective scaffold proteins IscU/Isu, but the N-terminus of the hHSC20 is clearly different from the J co-chaperones of fungi and most bacteria. The human protein contains an additional domain, which harbors two CXXC modules (C41/C44 and C58/C61). Their ability to coordinate a zinc ion in vitro results in zinc-finger-like structure. It was presented that the N-terminal cysteine motifs are important for hHSC20 function, perhaps because they bind a metal and stabilize a specific conformation of hHSC20 [50, 54]. Moreover, it was shown that human HSC20 protein could complement for its counterpart in yeast, Jac1, and interacted with its proposed human partners, hISCU and hHSPA9 (mortalin), which is the only mitochondrial Hsp70 protein in human. RNA interference-mediated depletion of hHSC20 specifically reduced the activities of both mitochondrial and cytosolic ISC-containing enzymes, which confirmed the conserved function of HSC20 in humans [54].

Available biochemical data [7] are consistent with the hypothesis that during the FeS cluster transfer process Jac1/mtHsp70 system utilizes the canonical Hsp70 client-binding cycle described above (Fig. 2). Jac1 co-chaperone binds to the Isu1 scaffold protein using its unique C-terminal client-binding domain [51, 55]. This interaction is highly specific as the Isu1 scaffold is the only client of Jac1 [6, 56]. In both the bacterial and mitochondrial systems, the C-terminal domain of Jac1 is directly responsible for Isu1 binding, with three hydrophobic residues playing a critical role in the interaction of Jac1 with Isu1 [51, 55, 57, 58] (Fig. 3). Substitution of these residues with alanine sharply reduces the interaction of Jac1 with Isu in vitro and severely compromises both cell growth and the activity of the FeS cluster containing enzymes in vivo. Hydrophobic residues of Jac1 consisting of leucines 105 and 109 on helix 6 and tyrosine 163 on helix 8 play a critical role in the Isu1 interaction both in vitro and in vivo. Yet, *jac1L105L109Y163* cells are viable. Only when replacements of the hydrophobic residues were combined with replacements of charged residues, a null phenotype was observed. Thus, the charged region does play a role, though not a critical one under laboratory conditions. Such a contribution of a number of residues toward the strength of protein–protein interaction across a binding interface has been observed previously for a variety of interacting proteins [59]. It is often the case that hydrophobic residues provide stability to the interaction, with the charged region providing specificity and directing the precise orientation of interacting partners [57]. The evolutionary conservation of the spatial orientation of both hydrophobic and charged patches across the binding interface of Jac1 and its bacterial and eukaryotic orthologs is consistent with such a mechanism.

Once Jac1–Isu1 complex is formed it interacts with partner mtHsp70. Transfer of the Isu1 client to the substrate binding domain of mtHsp70 and synergistic stimulation of the mtHsp70’s ATPase activity via the N-terminal J-domain of Jac1 are required for a stable mtHsp70–Isu1 interaction [53, 56, 60]. Residues L132, P133, P134, V135 and K136 of Isu1 are specifically recognized by mtHsp70 [6, 53]. In the ADP-bound form the Isu1 scaffold is stably associated with Hsp70 and in this configuration, the FeS cluster may be labilized and transferred toward apoproteins. This partial reaction is accompanied by the NEF (nucleotide exchange factor)-assisted exchange of ADP for ATP which then triggers the dissociation of Isu1 from mtHsp70. In contrast to the bacterial HscA which interaction with nucleotide is intrinsically transient, mtHsp70 has a high affinity for nucleotide, and demands nucleotide exchange factor, Mge1 (Fig. 2). Thus, whereas the bacterial and mitochondrial chaperone systems share critical features, they possess significant biochemical differences as well [56]. In its ATP-bound form the Hsp70 is ready for the next cycle of Jac1–Isu1 (or Hsc20–IscU) binding. Biochemical data, available for the bacterial Hsp70 system, shows that in the presence of ATP chaperones labilize FeS cluster from molecular scaffold, thus allowing the transfer of the cluster onto the recipient apo-protein [61, 62]. Bonomi and Vickery performed an elegant mechanistic studies on the catalysis of FeS cluster transfer from IscU[2Fe2S] by HscA/HscB chaperones in which they showed that HscA-mediated acceleration of [2Fe2S] cluster transfer exhibited an absolute requirement for both HscB and ATP. A mutant form of HscA lacking ATPase activity, HscA(T212V), was unable to accelerate cluster transfer, suggesting that ATP hydrolysis and conformational changes accompanying the ATP (T-state) to ADP (R-state) transition in the HscA chaperone are required for catalysis. Experiments carried out under conditions with limiting concentrations of HscA, HscB, and ATP further showed that formation of a 1:1:1 HscA–HscB–IscU2[2Fe2S] complex and a single ATP hydrolysis step are sufficient to elicit the full effect of the chaperones on the [2Fe2S] cluster. These results suggest that acceleration of iron–sulfur cluster transfer involves a structural change in the IscU2[2Fe2S] complex during the T into R transition of HscA accompanying ATP hydrolysis [63].

The first protein on the way to the recipient apo-protein can be the monothiol glutaredoxin 5 (Grx5), which interacts with mitochondrial specialized Hsp70, Ssq1, at a binding site different from that of Isu1. It was shown in vivo that Grx5 is an FeS protein and receives its FeS cluster from the Isu1 scaffold. The specific complex formation between Grx5 and the dedicated Hsp70 chaperone Ssq1 promotes the transfer of the FeS cluster synthesized on the scaffold protein Isu1 to target FeS apoproteins. The monothiol Grx5 enters the chaperone cycle by associating with Ssq1 at a specific binding site that is independent of that of Isu1, since both proteins can interact simultaneously with Ssq1 [41]. After dissociation of the trimeric complex Ssq1–Isu1–Grx5, holoGrx5 cooperates with the late-acting targeting factors of the ISC assembly machinery to deliver and assemble FeS clusters on target apoproteins. Grx5 is required for the maturation of mitochondrial [2Fe–2S] and [4Fe–4S] proteins, as well as of cytosolic FeS proteins. It precedes the function of the more specific ISC-targeting factors [41].

Grx5 is structurally well understood. It folds into a rigid body consisting of five alpha helices and four beta sheets. Purified monothiol glutaredoxins are capable of binding a bridging [2Fe–2S] cluster that is coordinated by two active-site cysteine residues (Cys67 on each Grx5 monomer) and two non-covalently bound glutathione molecules [64–67]. Formation of FeS cluster within Grx5 depends on the core ISC assembly machinery [41]. The Grx5-bound FeS cluster is labile and can be readily transferred to recipient apoproteins in vitro, consistent with FeS cluster transfer role of Grx5 [40, 68, 69]. In the bacterial ISC system, the presence of the Hsp70 HscA/HscB largely stimulates FeS cluster transfer from IscU to Grx5 [62]. FeS cluster binding to IscU is loosened up by ATP hydrolysis, favoring its transfer to Grx5 in the bacterial system [62, 70].

Recently it was presented that direct interaction between HSC20 and SDHB occurs and it requires presence of two L(I)YR motifs. In succinate dehydrogenase B, two L(I)YR motifs engage the ISCU–HSC20–HSPA9 complex to aid incorporation of three FeS clusters within the final structure of complex II [71]. One of the L(I)YR motifs appears in SDHB near the N-terminus, proximal to the first cysteines that ligate the [2Fe–2S] cluster, whereas the second is closer to the C-terminus and the cysteinyl ligands of the [4Fe–4S] and [3Fe–4S] clusters, in positions where binding of the chaperone–co-chaperone transfer apparatus can guide release of the cluster from holo-ISCU into the distal FeS binding sites of SDHB. The two L(I)YR motifs in SDHB are highly conserved throughout the eukaryotic and prokaryotic kingdoms, suggesting that these consensus sequences have significant functional importance. Biogenesis of SDHB may require binding of the orthologous co-chaperones to the L(I)YR motifs in organisms ranging from eukaryotes, including plants, to prokaryotes. Altogether, Maio and coworkers suggest that L(I)YR motifs are molecular signatures of specific recipient FeS proteins or accessory factors that assist FeS cluster delivery [71, 72].

## Evolution of the Hsp70 system

Mitochondria inherited most, but not all, of the components of ISC pathway from their endosymbiont ancestor. The scaffold (Isu1 in *S. cerevisiae*) and the specialized J-protein (Jac1 in *S. cerevisiae*) are present in all eukaryotes. However, the gene encoding HscA was either not inherited or lost early during evolution, as an *hscA* orthologue has not been found in any eukaryotic genome examined thus far. However, FeS cluster biogenesis is critical in all organisms. *SSC1*, the paralogue of *SSQ1*, encodes an abundant mtHsp70, which performs the remaining tasks of the ancestral protein, including transport of polypeptides across the mitochondrial inner membrane and protein folding as well as the maintenance of mtDNA [5]. Most eukaryotes, including humans, have a single, multifunctional mtHsp70. Only a subset of fungi, including *Saccharomyces cerevisiae*, also contains a highly specialized mtHsp70 involved in the essential process of iron–sulfur (FeS) cluster biogenesis, Ssq1. Ssq1 is encoded by a gene that arose through the duplication of an mtHSP70 gene in a common ancestor of *Candida albicans* and *S. cerevisiae* [6, 73] about 300 million years ago [74]. In contrast to Ssq1, Ssc1, a multifunctional Hsp70 interacts with various polypeptides including peptide containing the conserved LPPVK peptide loop of Isu1/IscU which is selectively recognized by Ssq1/HscA [4, 6, 53, 75–78].

All Hsp70s, including Ssc1 and Ssq1, require a J-protein co-chaperone. Ssc1 of *S. cerevisiae* is known to function with two J proteins: Pam18 and Mdj1 [79]. Neither Pam18 nor Mdj1 stimulates the ATPase activity of Ssq1 [56, 80], reflecting the fact that Ssq1 has lost the ability to perform the protein translocation and folding activities of Ssc1. Ssq1 functions with a single J protein, Jac1. The ATPase activity of Ssc1 is stimulated by Jac1, but much less effective than that of Ssq1, suggesting that Jac1 of *S. cerevisiae* has retained some residual ability to function with Ssc1 [6].

Extensive study determined that the existence of Ssq1 in fungal species correlates with structural and functional changes in the J domain of Jac1 [8]. Systematic analysis of the loop region of Jac1 proteins’ J domains reveals that it is shorter than the one present in either bacterial or human orthologs. It is suggested that only after a deletion occurred in *JAC1*, which reduced the size of the J domain, interaction with Ssc1 was weakened, but it did not affect interaction with Ssq1. The deletion event might have acted as an evolutionary ratchet, making reversal to the ancestral structure–function relations far more difficult, thus promoting forward co-evolution of Jac1 and Ssq1. Over time, reciprocal changes involving the sequence outside the loop region of Jac1 resulted in a highly specific and efficient interaction between Ssq1 and Jac1, forming a chaperone machinery tuned for functioning exclusively in FeS cluster biogenesis.

Recently it was shown that the mutational robustness of the Jac1 co-chaperone increased as it began partnering with the specialized Hsp70 Ssq1 upon its emergence through duplication of multifunctional Hsp70 Ssc1 [9]. Several, mechanisms could explain the higher mutational robustness of Jac1 that functions with specialized Ssq1. The simplest possibility is that Ssq1’s coevolution with Jac1 resulted in expansion of their binding interface, thus increasing the efficiency of their interaction. Such an expansion could in turn compensate for negative effects of HPD substitutions. As both Ssq1 and Jac1 are evolving at comparable rates, they could affect each other’s rate of amino acid substitution through mutual induction of compensatory changes. Another possible explanation of the higher tolerance of Jac1 for mutations is that by functioning with specialized Ssq1 it experienced relaxation of functional constraints [9]. In preduplication species, Jac1 competes with other J-protein co-chaperones Mdj1 and Pam18 for a common Hsp70 partner [7]. Such competition among co-chaperones in a crowded cell environment could constrain amino acid substitutions on their and Hsp70’s surfaces. Thus, lack of such competition could enable the increased rate of the Jac1 and Hsp70 sequence evolution.

## Functions of chaperones beyond the ATPase cycle

The cysteine desulfurase Nfs1 and the J-protein co-chaperone Jac1 bind to overlapping sites on Isu1, each containing the hydrophobic residues (Leu63, Val72, and Phe94) [81] (Fig. 3). Moreover, the same analysis showed that hydrophobic residues in the C-terminal region of Nfs1 (Leu479, and Met482) are involved in the interaction with the aforementioned hydrophobic patch. Consistent with this dual role, Jac1 and Nfs1 competed with each other for Isu1 binding in vitro, suggesting that their interactions with Isu1 are mutually exclusive and an ordered transition from cluster assembly to cluster transfer (Fig. 5).

**Fig. 4:**
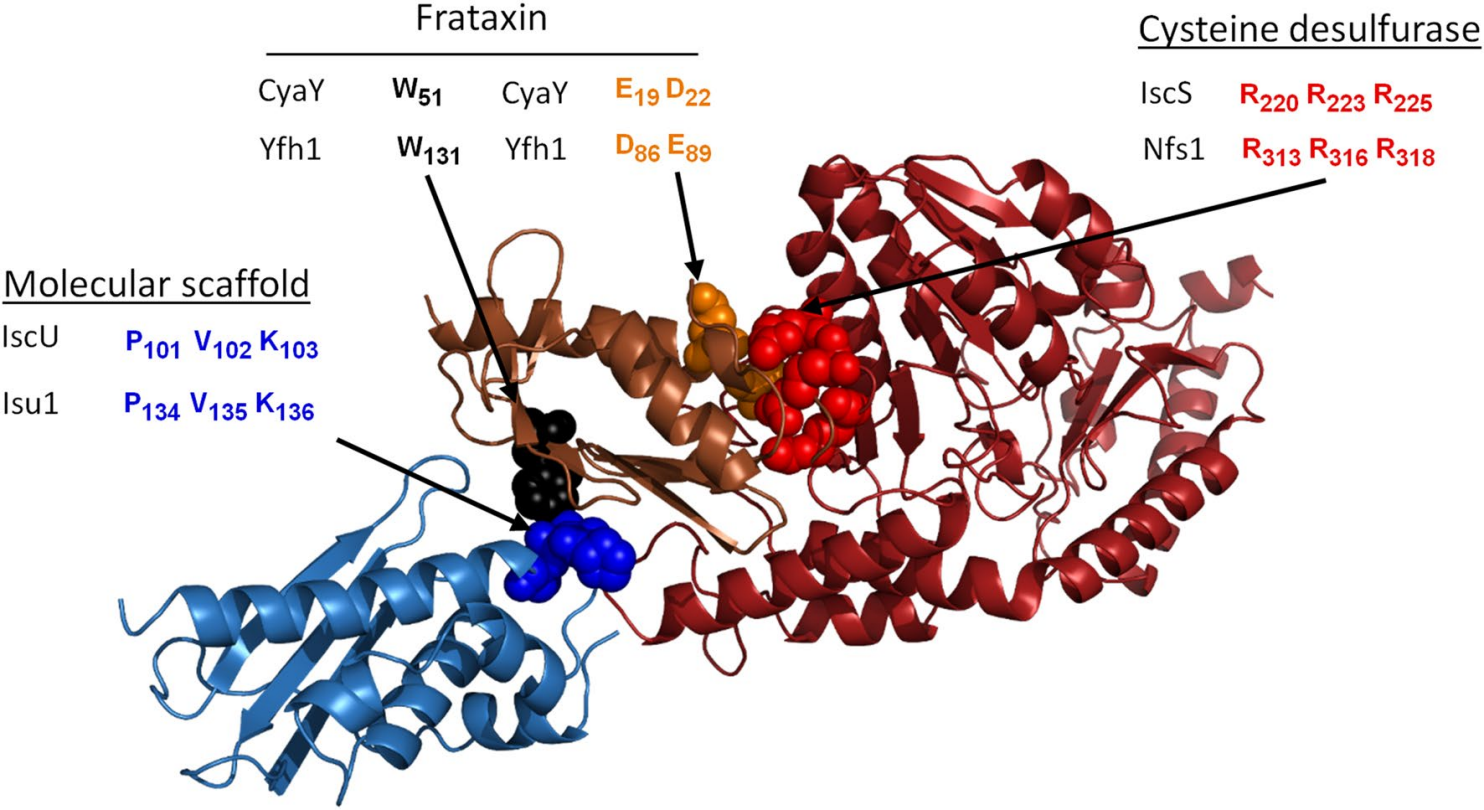
Structural model of bacterial FeS cluster assembly complex. PDB file of bacterial FeS assembly complex [100]. Residues predicted to be critical for frataxin interaction with the FeS cluster scaffold and cysteine desulfurase are listed for bacterial proteins (CyaY, IscU, and IscS) and their yeast orthologs (Yfh1, Isu1, and Nfs1, respectively), as indicated [60]

**Fig. 5:**
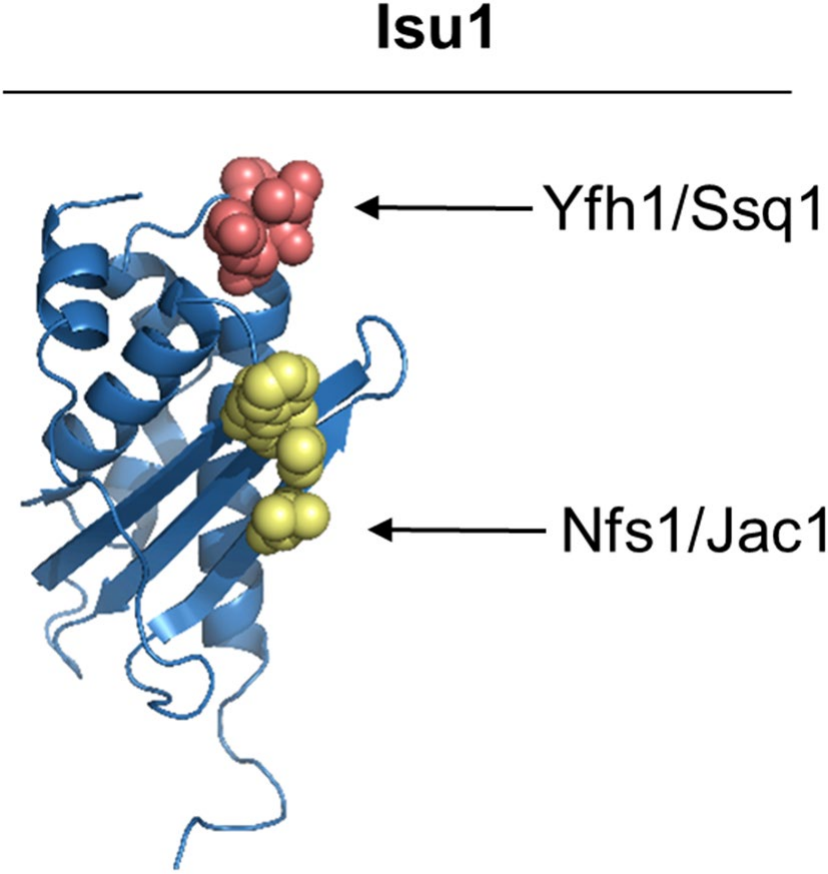
Homology model of Isu1 [81] with highlighted residues involved in Yfh1/Ssq1 (red) and Nfs1/Jac1 (yellow) complex formation [60]

Interestingly, the Yfh1 interaction with Isu involves the LPPVK sequence motif, which is also the key site for the interaction of Isu with Hsp70 Ssq1 (Figs. 4, 5). Coupled with our previous observation that Nfs1 and Jac1 binding to Isu1 is mutually exclusive due to partially overlapping binding sites, it can be proposed that such mutual exclusivity of cluster assembly factor (Nfs1/Yfh1) and cluster transfer factor (Jac1/Ssq1) binding to Isu1 has functional consequences for the transition from the assembly process to the transfer process, and thus regulation of the biogenesis of FeS cluster proteins [60] (Fig. 5). We propose that holo-Isu1 released from Nfs1(Isd11) by the action of Jac1 will also result in Yfh1 release, thereby exposing the LPPVK site for Hsp70 interaction. Once Ssq1 binds the LPPVK motif, Isu1 is “protected” against Yfh1 rebinding, even if Nfs1(Isd11) rebinds to Isu1 after dissociation of Jac1. Thus, the chaperones may play a regulatory role by controlling the flow of FeS clusters from the assembly complex to the recipient proteins. Further work is required to understand the dynamics of interactions between the cluster-bound and cluster-free forms of the scaffold and the interacting components involved in assembly and transfer.

Since FeS cluster biogenesis is essential, abolishing either step results in cell death. Partial impairment not only results in activation of Aft1/2 [82], but also an increase in the level of Isu1 [78, 83–85]. This increase is due to an increase in the stability of the Isu1 protein and its regulation occurs at the posttranslational level. The increase in Isu1 levels occurs when the functionality of components that act in either the assembly or transfer steps of the biogenesis process is reduced. This upregulation is specific to Isu1, as the levels of the other factors that function during cluster biogenesis are not affected [85]. In vivo and in vitro results demonstrate that the Lon-type protease of the mitochondrial matrix, Pim1, is responsible for degradation of Isu1. Its absence, but not the absence of other mitochondrial proteases, in vivo results in a dramatic increase in Isu1 levels [86].

J co-chaperone, Jac1, is one of two proteins which has a role in the protection of Isu1 against degradation by Lon-type protease of the mitochondrial matrix, Pim1. The another protein is Nfs1. These two factors, Jac1 and Nfs1, whose site of interaction on Isu1 overlap (Leu63, Val72, and Phe94), are capable of protecting Isu1 from degradation both in vivo and in vitro [86, 87]. The alternations of these residues to the polar residue, serine (Isu1 LVF_SSS) significantly decreased Isu’s susceptibility to degradation [86]. These results underscore the importance of the Jac1–Isu1 interaction for both driving and coordinating FeS cluster biogenesis.

## Future perspectives

The proteins involved in the mitochondrial process of FeS cluster biogenesis are highly evolutionarily conserved across Eukaryotes. Orthologs of yeast proteins involved in this process are readily identifiable in humans, underscoring the biological importance of the FeS cluster biogenesis process [13]. This conservatism also makes experimental results obtained for yeast proteins easily convertible to mammalian and human systems.

In mammals, the loss of individual components of mitochondrial FeS cluster biogenesis pathway is lethal during early embryonic development. Thus, it is not surprising that mutations in FeS cluster biogenesis genes may cause severe disorders in humans, including such important diseases as Friedreich’s ataxia, hereditary myopathy with lactic acidosis and several different sideroblastic anemias (SA). Many of them are fatal, sometimes already in early childhood. Mutations associated with human disorders were found in genes encoding frataxin (Yfh1 in fungi) [88, 89]; FeS scaffold protein (Isu1 in fungi) [90–93], ferredoxin (Yah1 in fungi) [94] cysteine desulfurase accessory protein (Isd11 in fungi) [95], mitochondrial monothiol glutaredoxin (Grx5 in fungi) [96, 97] and mitochondrial Hsp70 (mtHsp70/Ssq1 in fungi) [98]. Biochemical study of the fungal orthologs of all these disease-associated genes can improve our basic knowledge of the mechanistic biochemical aspects of mitochondrial FeS cluster biogenesis process. Such approach will help us to understand the molecular background of human pathologies associated with this critical metabolic pathway. Moreover, in the long run, some of our findings could turn out crucial for the development of new knowledge-based therapies.

Another important and unresolved problem is associated with the emergence of the specialized mtHsp70 by gene duplication. What is the functional significance of this phenomenon? In other words, does FeS protein biogenesis in which the new specialized protein participates is qualitatively different from the analogous process in which the multifunctional protein participates? In most cases, a single Hsp70 interacts with many J-proteins, each of them delivering specific substrates to their Hsp70 partner. Yet, in some cases, a more specialized Hsp70, functions with a single J-protein partner [99]. Little is known about what drives specificity of the Hsp70 interaction with a given J-protein co-chaperone, or how J-protein recognizes one but not another Hsp70 partner. Also, the molecular mechanisms behind productive Hsp70/J-protein substrate interactions are poorly understood. To answer these questions additional structural studies, especially characterization of the various protein complexes and the different conformational states of the chaperones, are needed to provide a three-dimensional understanding of the nature of the interactions and how these regulate chaperone activity.

Finally, an interesting question is how Hsp70 binding affects the structure and dynamics of Isu1? In other words, whether any Isu conformational changes occur upon Hsp70 interaction that influences its ability to coordinate FeS cluster. Current evidence suggests that the primary role of the chaperones is likely to facilitate cluster release to apo-acceptor proteins, but the exact nature of the effects on FeS-scaffold complexes and how the chaperones bring about these changes remain unknown.
